# Outcome of colon cancer initially presenting as colon perforation and obstruction

**DOI:** 10.1186/s12957-017-1228-y

**Published:** 2017-08-25

**Authors:** Tsung-Ming Chen, Yen-Ta Huang, Guan-Chyuan Wang

**Affiliations:** 1 Department of Surgery, Buddhist Tzu Chi General Hospital, Hualien, Taiwan; 2 Department of Colorectal Surgery, Buddhist Tzu Chi General Hospital, 707, Section 3, Chung Yang Road, Hualien, 970 Taiwan; 3 Surgical Intensive Care Unit, Buddhist Tzu Chi General Hospital, 707, Section 3, Chung Yang Road, Hualien, 970 Taiwan; 4 School of Medicine, Tzu Chi University, Hualien, Taiwan; 5 Department of Pharmacology, Hualien, Taiwan; 6 Department of Neurosurgery, Neuro-Medical Scientific Center, Buddhist Tzu Chi Hospital, 707, Section 3, Chung Yang Road, Hualien, 970 Taiwan

**Keywords:** Colon cancer, Perforation, Obstruction

## Abstract

**Background:**

Emergency complications of colon cancer include perforation and obstruction which were recognized as poor prognostic factors. Few studies have directly compared the outcomes of these two groups. In this study, we evaluated mortality and morbidity in patients with colon cancer initially presenting as perforation and obstruction.

**Methods:**

Newly diagnosed colon cancer cases initially presenting with perforation or obstruction at Tzu Chi General Hospital, Hualien, Taiwan, between 2009 and 2015 were included. Cases of iatrogenic perforation or perforation sites far away from the tumor sites and rectal (< 15 cm from the anal verge) cancer were excluded. Progression-free survival, local recurrence rate, distant metastasis rate, and overall survival were the evaluated outcomes.

**Results:**

Eighty-one patients met the selection criteria; 23 and 58 patients had perforation and obstruction, respectively, as the initial symptom. The median age was 72 years. The median tumor stage was stage IIIB. The 1-year and 3-year survival rates were 83.7 and 59.7%, respectively. The perforation group (PRG) and obstruction group (OBG) did not differ significantly in intensive care unit (ICU) stay rate (*p* = 0.147), sex (*p* = 0.45), comorbidities (heart, liver, and renal diseases and diabetes mellitus), median stage (*p* = 0.198), and overall survival (*p* = 0.328). However, PRG had a higher age at diagnosis (74 vs. 64 years, *p* = 0.037), a higher APACHE II score (12 vs. 7, *p* = 0.002), lower disease-free survival (*p* = 0.001), a higher recurrence rate (56.5 vs. 19%, *p* = 0.002), a higher distant metastasis rate (39.1 vs. 13.8%, *p* = 0.015), and a higher local recurrence rate (43.5 vs. 5.2%, *p* < 0.001) than did OBG. OBG had a higher two-stage operation rate (46.6 vs. 17.4%, *p* = 0.022). After adjustment for the tumor stage, comorbidity (chronic renal disease), body mass index (BMI), and adjuvant chemotherapy or radiotherapy in multivariate statistics, PRG had lower disease-free survival (*p* = 0.005) than OBG but overall survival was identical.

**Conclusion:**

For colon cancer initially presenting as perforation or obstruction, the PRG had poorer progression-free survival, a higher local recurrence rate, and a higher distant metastasis rate than did OBG. Overall survival did not differ between these two groups.

## Background

Emergency complications of colon cancer include perforation and obstruction, and 15–40% of patients with colorectal cancer initially present these conditions [1]. Colon cancer with perforation comprises 3–10% of the initial presentation of colon cancer, and that with obstruction comprises 8–40% [1–4]. These complications have indicated that poor prognostic factors influence outcomes [5–11]. Furthermore, in the European Society for Medical Oncology (2012) and National Comprehensive Cancer Network (2014) guidelines, colon cancer with perforation or obstruction is considered a poor prognostic factor along with T4 primary tumors, inadequately sampled nodes, lymphatic vessel invasion, and perineural invasion [12, 13]. However, few studies have directly compared the outcomes of perforation with those of obstruction in colon cancer. Moreover, most studies have pertained to rectal cancer, which has a different pathophysiology from that of colon cancer. In this study, we evaluated mortality and morbidity in patients with colon cancer initially presenting as perforation and obstruction.

## Methods

### Patients

**Fig. 1:**
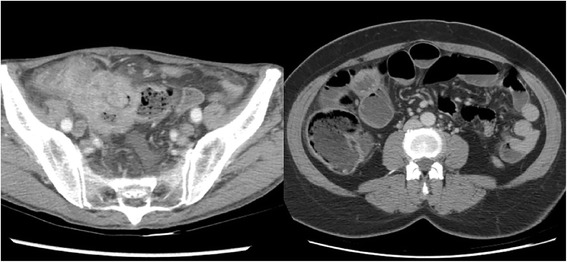
The image on the left depicts colon perforation because of sigmoid colon cancer. The image on the right depicts bowel distension caused by complete obstruction of the transverse colon

We retrospectively reviewed medical records from 2009 to 2015. Newly diagnosed cases of colon cancer initially presenting as perforation or obstruction in Tzu Chi General Hospital, Hualien, Taiwan, were included. We reviewed and recorded the initial presenting symptoms, the length of intensive care unit (ICU) stay, imaging results, intraoperative results, operative methods, final pathological reports, tumor stage, APACHE (Acute Physiology and Chronic Health Evaluation) II score, adjuvant therapy, body mass index (BMI), comorbidities, local recurrence, distant metastasis, and survival time. Cases of iatrogenic perforations, distant perforation sites, and rectal cancers (< 15 cm from the anal verge) were excluded. Patients who did not return to the outpatient department after discharge were defined as lost follow-up and also excluded. The perforation group (PRG) included patients with a colon perforation at the primary cancer site, which was confirmed using images, pathological findings, and operative records (Fig. 1). Patients in the obstruction group (OBG) was assigned through clinical and radiological and intraoperative findings (complete obstruction) (Fig. 1). Local recurrence was defined as recurrent tumors in the original tumor bed. Distant recurrence was defined as recurrent tumors outside the peritoneal cavity. Overall survival was defined as the duration from operation to death or the last follow-up. Disease-free survival was defined as the duration from operation to cancer recurrence. Stage operation was defined as having undergone diversion surgery (colostomy or ileostomy) followed by an arranged definite surgery on another day.

### Statistics

The Mann–Whitney *U* test was used to calculate univariate continuous variables. The chi-squared test was used to calculate univariate categorical variables. The Kaplan–Meier curves and Cox proportional hazard models were used for survival analysis. We used IBM SPSS 22 (IBM Corporation, Armonk, NY, USA) for statistical analysis, and *p* < 0.05 was considered statistically significant.

## Results

In our hospital, 527 patients were diagnosed with colon cancer from 2009 to 2015. Among the 81 patients who met the inclusion criteria, 23 patients had perforation and 58 patients had obstruction as the initial symptom. The perforation and obstruction rates were 4.3 and 11%, respectively, in all colon cancers. The median age was 72 years (19–92 years), and the median tumor stage was stage IIIB (stages I–IVB). The male-to-female ratio was 51:30. The sigmoid colon was the most common tumor location (28 patients, 35%; Table 1), and 25 patients (30.9%) were admitted to ICUs. The median length of ICU stay was 3.5 days (1–83 days). The 1-year and 3-year survival rates were 83.7 and 59.7%, respectively.

**Table 1:** Distribution of colon cancer in the patients

Location	Patients (%)
Sigmoid colon	28 (35)
Descending colon	13 (16)
Transverse colon	13 (16)
Ascending colon	8 (9.9)
Cecum	8 (9.9)
Rectal-sigmoid junction	5 (6)
Appendix	2 (2.5)

Distribution of colon cancer with perforation and obstruction in our patients

**Fig. 2:**
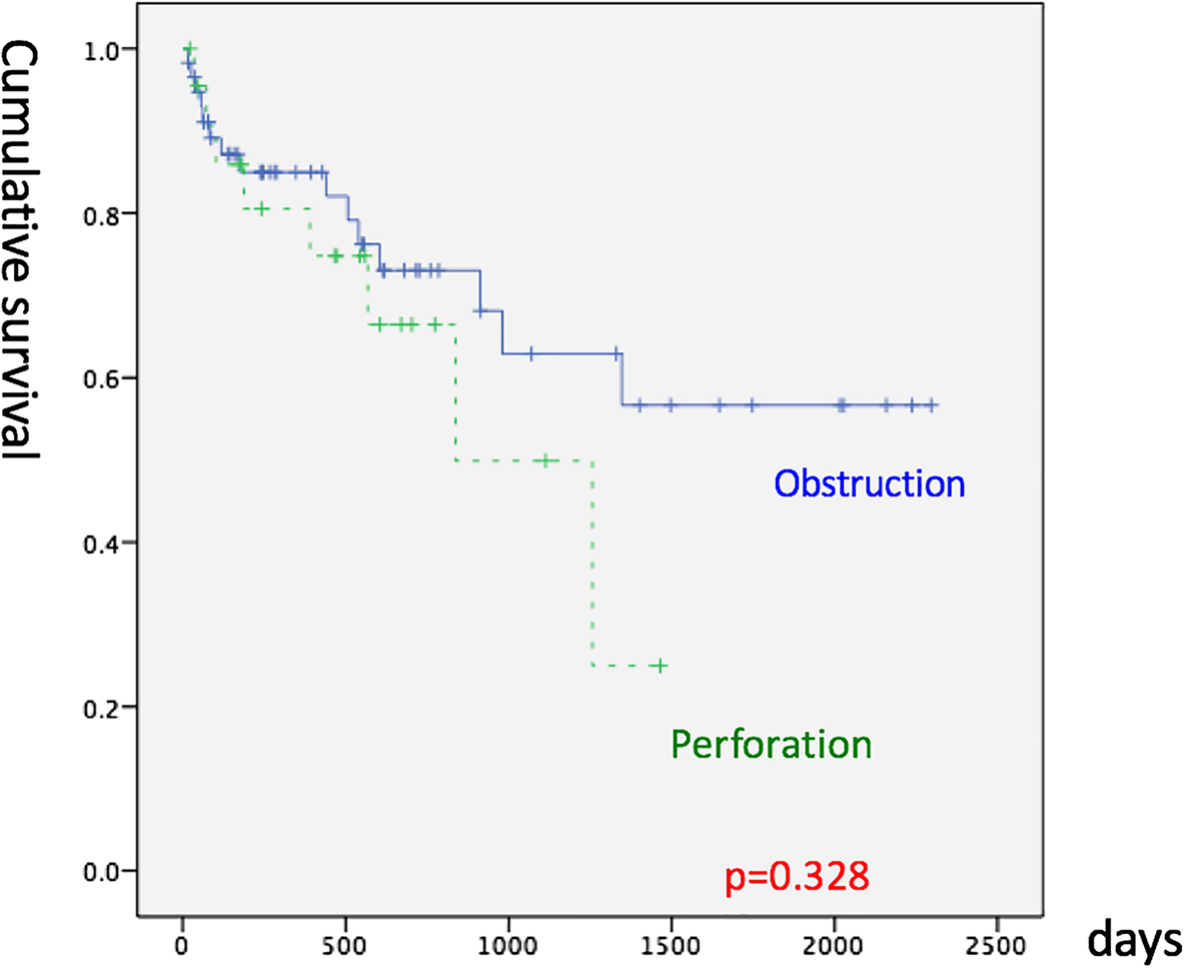
Comparison of overall survival between PRG and OBG. PRG colon cancer initially presenting as perforation, OBG colon cancer initially presenting as obstruction

**Fig. 3:**
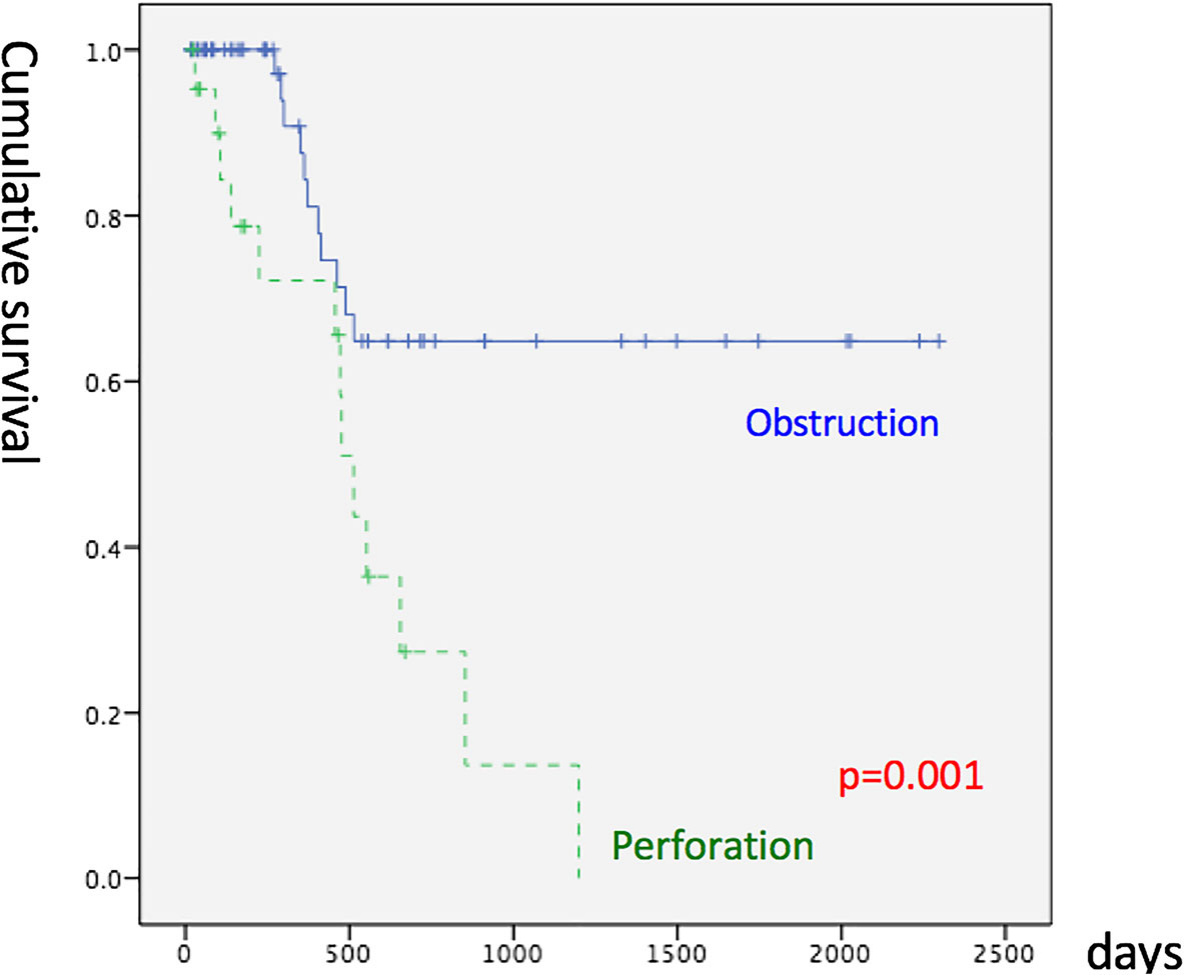
Comparison of disease-free survival between PRG and OBG. PRG colon cancer initially presenting as perforation, OBG colon cancer initially presenting as obstruction

The length of ICU stay, sex, comorbidities (heart, liver, and renal diseases and diabetes mellitus), median stage, and overall survival did not differ significantly between the two groups (Table 2; Fig. 2). However, the age at diagnosis was higher in OBG (74 vs. 64 years, *p* = 0.037), and OBG also had a higher APACHE II score (12 vs. 7, *p* = 0.002), a higher cancer recurrence rate (56.5 vs. 19%, *p* = 0.002), a higher distant metastasis rate (39.1 vs. 13.8%, *p* = 0.015), a higher local recurrent rate (43.5 vs. 5.2%, *p* = 0.022), and a poorer disease-free survival rate (*p* = 0.001; Table 2; Fig. 3). OBG had a higher two-stage operation rate (46.6 vs. 17.4%; Table 2) than did PRG. In multivariate statistics, after adjustment for the tumor stage, comorbidity (chronic renal disease), BMI (representing nutrition status), and treatment (chemotherapy or radiotherapy), PRG had a poorer disease-free survival (*p* = 0.005) than did OBG but the overall survival was identical in these two groups (Tables 4 and 6). A high tumor stage was associated with poor survival (*p* = 0.007). High BMI was also associated with poor survival (hazard ratio 2.26, *p* = 0.066; Tables 3 and 4). Patients receiving adjuvant chemotherapy or radiotherapy had improved survival (hazard ratio 0.202, *p* = 0.002) (Tables 3 and 4). The comparison between the results for PRG and OBG is listed in Table 5.

**Table 2:** Comparison between the two groups

	PRG	OBG	*p*
Age (years old) (median)	64	74	0.037
Sex (male to female)	13:10	38:20	0.45
ICU stay	39.1%	27.6%	0.147
APACHE II score (median)	12	7	0.002
BMI (median)	24.2	22.3	0.114
Heart disease	13%	6.9%	0.562
Liver disease	0%	5.2%	0.554
Diabetes	39.1%	20.7%	0.1
Chronic kidney disease	4.3%	13.8%	0.434
Tumor recurrent rate	56.5%	19%	0.002
Median stage	IIIC	IIIB	0.198
Distant metastasis rate	39.1%	13.8%	0.015
Local recurrent rate	43.5%	5.2%	<0.001
Two-stage operative rate	17.4%	46.6%	0.022

Univariate analysis to compare PRG and OBG

*PRG* colon cancer initially presenting as perforation, *OBG* colon cancer initially presenting as obstruction

**Table 3:** Factors affecting survival

	Hazard ratio	*p*
Age	1.005	0.752
Sex	0.870	0.517
Stage		
I + II	1	0.031
III	0.723	
IV	3.033	
ICU stay	1.768	0.200
APACHE II score	1.057	0.194
BMI		
< 18.5	1	0.039
18.5–24	1	
> 24	2.41	
Heart disease	1.31	0.718
Liver disease	0.827	0.855
Diabetes	1.429	0.472
CKD	2.348	0.092
Adjuvant chemotherapy/radiotherapy	0.233	0.001
Right-side colon cancer	0.848	0.695

In univariate analysis, BMI > 24 was associated with poor survival. CKD induced poor survival. Receiving adjuvant chemotherapy or radiotherapy was associated with better survival

*CKD* chronic kidney disease, *BMI* body mass index

**Table 4:** Risk and protective factors

	Hazard ratio	*p*
Perforation	1	0.149
Obstruction	1.961	
Stage		
I + II	1	0.007
III	1.154	
IV	4.901	
CKD	1.496	0.489
BMI	2.260	0.066
Adjuvant chemotherapy/radiotherapy	0.202	0.002

In the multivariate analysis of overall survival, tumor stage was a risk factor, whereas receiving adjuvant chemotherapy or radiotherapy was a protective factor. High BMI was associated with poor survival. Survival rates in perforation or obstruction were not significantly different

*CKD* chronic kidney disease, *BMI* body mass index

**Table 5:** Comparison between the results for PRG and OBG

	PRG	OBG
Overall survival	No significant difference	
Disease-free survival	Poorer	Better
Distribution	Older age	
	Higher APACE score	
Recurrent rate	Higher	Lower
Local recurrent rate	Higher	Lower
Distant metastasis rate	Higher	Lower
Two stage operation rate	Lower	Higher

Summary of comparison between the RPG and OBG

*PRG* perforation group, *OBG* obstruction group

## Discussion

**Fig. 4:**
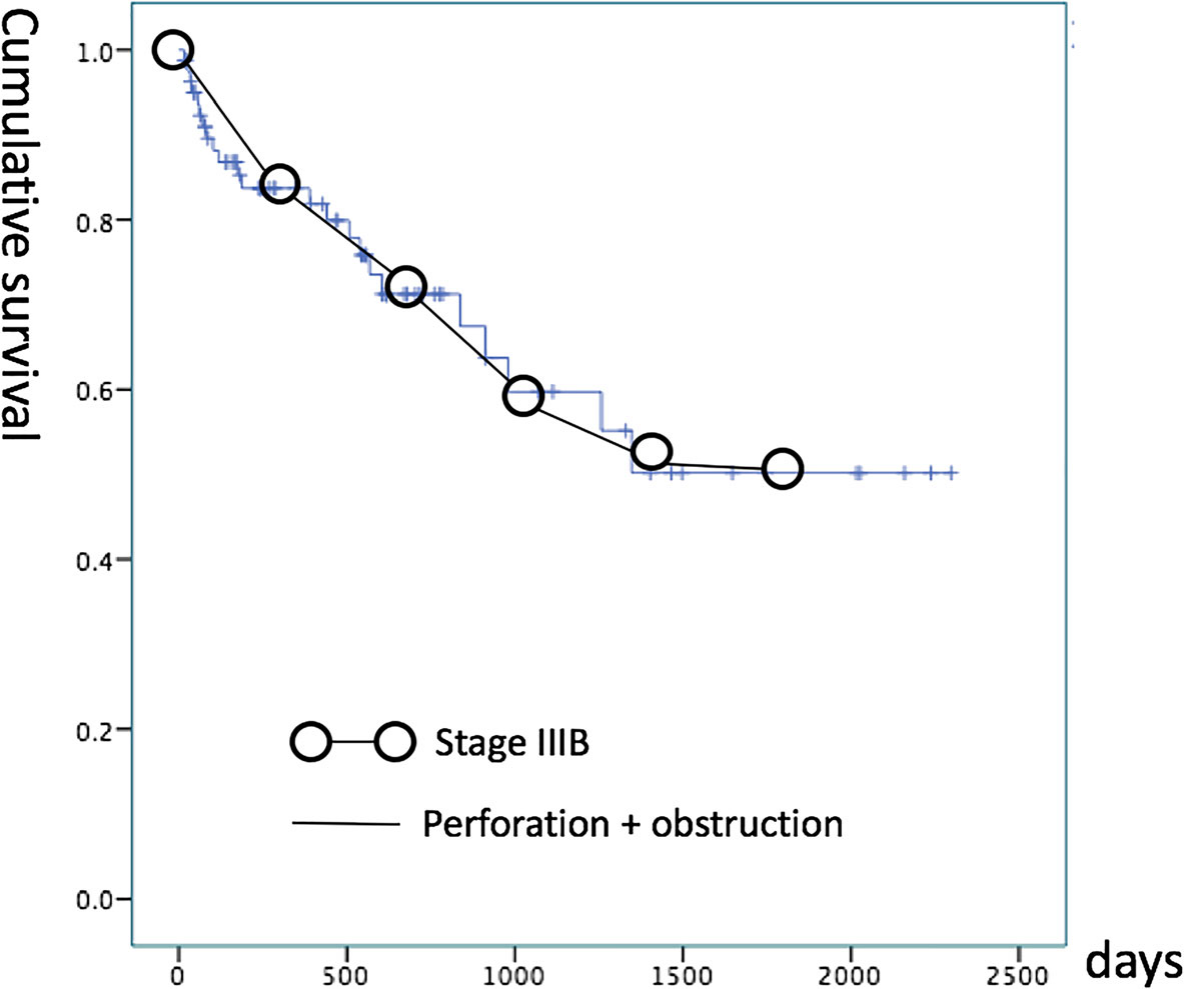
The survival curves of stage IIIB colon cancer and complicated (with perforation or obstruction) colon cancer are extremely similar. The overall 1-year survival rate was 83.7% and 3-year survival rate was 59.7% in colon cancer with perforation and obstruction. In comparison, the overall 1-year and 3-year survival rates were 83.4 and 59.3%, respectively, in stage IIIB colon cancer. The survival curve of stage IIIB colon cancer was plotted on the basis of the seventh edition of the American Joint committee on Cancer cancer staging manual and the future of TNM

Emergency colorectal cancer surgeries are associated with poor outcomes [14]. Obstruction and perforation are the two major factors. Chen et al. demonstrated that neoplastic bowel obstruction, but not bowel perforation at the tumor site, was associated with poor survival [9]. Banaszkiewicz et al. reported increased rates of complications and mortality in these patient groups [15]. Ho et al. reported that bowel obstruction and perforation were associated with poor disease survival in colorectal cancer [16]. In our study, colon cancer with perforation or obstruction showed an overall 1-year survival rate of 83.7% and a 3-year survival rate of 59.7%. We found that the survival curve was very similar to that of stage IIIB colon cancer (Fig. 4) [17]. The results can be explained by the following reasons: (1) increased age at diagnosis, (2) debility, (3) increased operative mortality, (4) advanced stage of disease at presentation, and (5) association with comorbidities (sepsis and acute kidney injury) [3, 18–20].

Our study focused on the comparison between colon cancer with perforation and that with obstruction. We defined the diagnoses of obstruction and perforation. We included patients with colon cancer and excluded patients with rectal cancer. Our results revealed that the overall survival did not differ significantly between obstruction and perforation in colon cancer. However, colon cancer with perforation had a poorer progression-free survival rate, a higher local recurrence rate, and a higher distant metastasis rate compared with colon cancer with obstruction (Table 6). Few studies have directly compared the prognosis of perforation and obstruction in colon cancer, and some studies did not offer clear definitions. Chen et al. compared complete bowel obstruction with bowel perforation at the site of tumor [9]. The results revealed that bowel obstruction had poorer overall survival than did perforation. However, this study also involved patients with rectal cancer. Although Alvarez et al. indicated that the perforation group had a higher postsurgical mortality rate than did the obstruction group (29 vs. 11%, respectively), this study also included patients with rectal cancer [7]. Biondo et al. published a study which might be the most specific study in comparing the survival rates between obstruction and perforation in colon cancer [1]. Similarly to our study, this study excluded patients with rectal cancer and included 236 patients. The results revealed that tumor recurrence and overall survival did not differ significantly between patients with obstruction and those with perforation in colon cancer. However, the perforation group included patients who had bowel perforation because of distension (usually caused by obstruction). In our study, we only included bowel perforation at the tumor sites in the perforation group.

**Table 6:** Disease-free survival comparison

	Hazard ratio	*p*
Perforation	1	0.005
Obstruction	3.261	
Stage		
I + II	1	0.952
III	1.076	
IV	0.887	
Adjuvant chemotherapy/radiotherapy	2.157	0.318

In multivariate analysis of disease-free survival, OBG had poorer disease-free survival than PRG

*OBG* obstruction group, *PRG* perforation group

In our study, the obstruction group had a higher two-stage operation rate (46.6%) than did the perforation group. The perforation group also showed a two-stage operation rate of 17.4%. The reasons for performing two-stage operations might be as follows: (1) to allow complete resuscitation before surgery, (2) presence of peritonitis or severe sepsis, and (3) bowel distension with fecal contamination, which increases surgical difficulty. However, recent studies have suggested that one-stage curative emergency resection had similar perioperative morbidity, mortality, and long-term survival compared with two-stage operations [4, 11, 21]. However, in patients with compromised immunity, malnutrition, septic shock, or severe comorbidities (severe cardiopulmonary disease), two-stage operations were an option [20].

## Conclusions

For colon cancer initially presenting as perforation or obstruction, the survival curve resembles stage IIIB colon cancer (3-year survival rate, 59.7 vs. 59.3%). Colon cancer with perforation had poorer progression-free survival, a higher local recurrence rate, and a higher distant metastasis rate compared with that with obstruction. The overall survival was identical. High tumor stage and high BMI were associated with poor survival. Patients who had received adjuvant chemotherapy or radiotherapy showed improved survival. We found a high rate of multiple-stage operations in our patient group, particularly in OBG. Further operative decisions might be adjusted to a one-stage curative tumor resection according to the current trend.

## Abbreviations

APACHE II score: Acute Physiology and Chronic Health Evaluation II score
BMI: Body mass index
ICU: Intensive care unit
OBG: Colon cancer with obstruction
PRG: Colon cancer with perforation
